# Size-Dependent Raman Shifts for nanocrystals

**DOI:** 10.1038/srep20539

**Published:** 2016-04-22

**Authors:** Yukun Gao, Xinmei Zhao, Penggang Yin, Faming Gao

**Affiliations:** 1 Key Laboratory of Bio-inspired Smart Interfacial Science and Technology of Ministry of Education, School of Chemistry and Environment, Beihang University, Beijing 100191, China; 2 Department of Applied Chemistry, Yanshan University, Qinhuangdao 066004, P. R. China

## Abstract

Raman spectroscopy is a very sensitive tool for probing semiconductor nanocrystals. The underlying mechanism behind the size-dependent Raman shifts is still quite controversial. Here we offer a new theoretical method for the quantum confinement effects on the Raman spectra of semiconductor nanocrystals. We propose that the shift of Raman spectra in nanocrystals can result from two overlapping effects: the quantum effect shift and surface effect shift. The quantum effect shift is extracted from an extended Kubo formula, the surface effect shift is determined via the first principles calculations. Fairly good prediction of Raman shifts can be obtained without the use of any adjustable parameter. Closer analysis shows that the size-dependent Raman shifts in Si nanocrystals mainly result from the quantum effect shifts. For nanodiamond, the proportion of surface effect shift in Raman shift is up to about 40%. Such model can also provide a good baseline for using Raman spectroscopy as a tool to measure size.

Raman spectroscopy is a powerful approach to the investigation of properties of semiconductor nanocrystals because the significant variations in Raman frequency with decreasing the size of materials can be easily detected^1^,^2^,^3^. In order to study the size-dependence of Raman frequency of nanocrystals, some theoretical phonon models have been developed. Most of the theoretical understanding of phonon modes in nanocrystals is based on the continuum dielectric models^4^,^5^,^6^,^7^,^8^. However, all dielectric models are only valid in the long-wavelength limit because one of their basic assumptions is that the material is homogeneous. When the size of nanocrystals is small, the continuum dielectric models are intrinsically limited. The calculated frequency shift of Raman spectra compare well with the experiment for larger size but failed in the range of a few nanometers. Although *ab initio* calculations appear to be a sophisticated method for the investigation of the Raman spectra^9^, one of the major difficulties of the microscopic modeling of phonon modes in nanocrystals is its computational intensity. For most nanosystems containing a few thousands atoms, *ab initio* calculations are still too laborious to be practical. To resolve these issues, a few useful phenomenological formulae for the size effect on Raman red shifts in semiconductor nanocrystals were developed^10^,^11^.

It should be noted that the previous models could replicate the experimental results broadly at the cost of certain adjustable parameters. A direct relation without any adjustable parameters between the Raman shift and the crystal size is still not available. As a rapid and nondestructive measure of the nanocrystal size, more accurate model for size-dependent Raman spectra is greatly needed^12^,^13^. In this work, we provide a simple relationship of the size-dependent Raman shifts based on the Kubo theory. In particular, it is found that surface atoms in nanocrystals play an important role in determining Raman shifts.

The energy levels in molecules or nanocrystals include the energy levels of electrons and nuclear motion. The rotational and vibrational energy levers are due to the motion of the nuclei. In general, energy level differences between electronic states are larger, differences between vibrational levels are intermediate, and differences between rotational levels are smallest in comparison^14^. The electronic states of nanocrystals differ markedly from those of the bulk materials due to quantum size effects(QSE) first described by Kubo^15^. According to Kubo theory, the electronic energy level shift *δ* increases with decreasing particle size, which is given by *δ* = (*m*_e_*e*^4^/2*ћ*^2^)(4*N*)^−1/3^ eV, where *N* is the number of atoms in nanocrystals^15^. Such quantum size effects often result in an absorption edge blue shift in ultraviolet-visible absorption spectra.

In rovibronic coupling, electron transitions are simultaneously combined with both vibrational and rotational transitions. To each electronic state *E*_e_ there are many bound vibrational states of energy *E*_v_, and each vibrational level has its own set of rotational levels *E*_r_. The total energy can be written as *E* = *E*_e_ + *E*_v_ + *E*_r_. The typical electronic frequencies, *ω*_e_ lie in the ultraviolet-visible region of spectrum^14^. The typical vibrational frequencies, *ω*_v_ and rotational frequencies, *ω*_r_ lie in the infrared region of spectrum. According to Herzberg’ theory^16^,


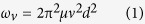



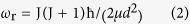


where *μ* is the reduced mass, *ν* is frequency, *d* is internuclear spacing.

Raman spectroscopy is an important tool in gathering information about molecular and crystal vibrational and rotational properties. Raman spectra, being similar to the ultraviolet-visible absorption case, can show significant changes when the size of investigated crystals goes down to the nanometer scale. Wichmann^17^ suggested a crude order of magnitude which is estimate as: *E*_r_ = (*m*_e_/M)*E*_e_, where *m*_e_ is the electronic mass, M is the reduced mass of nuclei. The mass of a nucleus is large compared to the mass of an electron, and the quantity M is of the order of a proton mass M_p_^16^. For Raman shifts resulting from the quantum effect of nanocrystals, like *Kubo effect* in QSE, we can propose an expression as


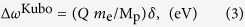


where *Q* = 2(*Z*_*c*_/*N*_*c*_), is referred to as an effective number of electrons bounding in a bond, where *Z*_*c*_ and *N*_*c*_ are the valence and coordination of the cation.

A semiconductor nanocrystal is composed of surface atoms and inside atoms. The bond length and coordination number of surface atoms and inside atoms are different. The total shift in Raman frequencies is due to the competing influences of surface atom under-coordination and Bond-length change. In our opinions, Raman shifts resulting from of nano effects may be resolved into contributions from the two types of bonds, surface bonds and inside bonds. We define the atoms with imperfect coordination number as the surface atoms^15^. The fraction of surface bonds composing the nanocrystal, *F*^surface^ equals the ratio of the number of surface bonds to the total number of bonds in the nanocrystal. The size-dependent Raman shift of nanocrystal with a given diameter D is expressed as


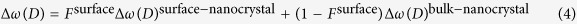


where Δ*ω*(*D*)^surface-nanocrystal^ is Raman shift of a hypothetical nanocrystal (denoted as “surface-nanocrystal”) whose atomic coordination number and bond length are entirely *N*_c_^surface^ and *d*^surface^ respectively. Δ*ω*(*D*)^bulk-nanocrystal^ is Raman shift of a hypothetical nanocrystal (denoted as “bulk-nanocrystal”) whose atomic coordination number and bond length are entirely *N*_c_^bulk^ and *d*^bulk^ respectively.

For a semiconductor nanocrystal, the under-coordination of surface atoms, *N*_c_^surface^ has a great impact on its vibrational frequency^9^. The size of such type impact can be quantitatively described by Eq.3. On the other hand, the bond length of surface atoms, *d*^surface^ is also different with that of bulk, *d*^bulk^. Eq. 1 and Eq. 2 show that the bond-length change may result in vibrational and rotational frequency shifts. Thus, the Raman shift of “surface-nanocrystal” can be expressed as


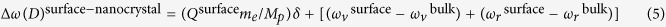


where *Q*^surface^ = 2(*Z*_c_/*N*_c_^surface^), *ω*_v_^surface^ and *ω*_v_^bulk^ represent the vibrational energy of the surface bond and the bulk phase, respectively. *ω*_r_^surface^ and *ω*_r_^bulk^ represent the rotational energy of the surface bond and the bulk phase, respectively. Since the rotational energy is far less than the vibrational energy, Eq. 5 may be simplified as:


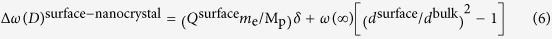


where *ω*_v_^bulk^ is taken as the Raman peak position of bulk, *ω*(*∞*). Obviously, Raman shift for a “bulk-nanocrystal” can be expressed as


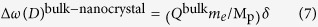


where *Q*^bulk^ = 2(*Z*_*c*_/*Nc*^bulk^). Thus, the size-dependent Raman shift of nanocrystal is expressed as


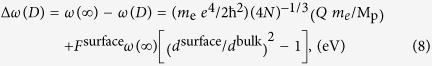


where *ω*(*D*) is the size-dependent Raman frequency, *Q* = *F*^surface^*Q*^surface^* *+* *(1 − *F*^surface^)*Q*^bulk^.

According to the method in ref. 15, the number of surface atoms and the length of bond at the surface of the semiconductor nanocrystals can be obtained using the Material studio program of Accelrys Inc. The geometry optimization by first principles calculations was carried out using the GGA approach by the Perdew-Burke-Ernzerhof (PBE) exchange correlation functional of density functional theory with the Material studio^18^. The calculations were performed by using an energy cutoff of 380 eV with the plane wave basis set. Brillouin zone (BZ) integration was performed using the special *k*-point Monkhorst-Pack sampling scheme. The optimized bond lengths of the diamond, Si, CdS, CdSe, InP, InAs single crystals, *d*^bulk^ are 1.531, 2.33, 2.561, 2.661, 2.57 and 2.648 Å, respectively. The obtained bond lengths of surface bonds of these nanocrystals, *d*^surface^ are 1.545, 2.319, 2.519, 2.630, 2.538, 2.60 Å, respectively.

The first-order Raman frequency *ω*(*∞*) of Si, CdS, CdSe, InP, and InAs single crystals are 520.5, 305, 212, 347 and 241.5 cm^−1^, respectively. Reliable values on Raman shifts of Si, CdS, CdSe, InP, and InAs nanocrystals are available.^2^,^12^,^13^,^19^,^20^,^21^,^22^,^23^. Here we carried out the systematic calculations of these nanocrystals using this proposed method. Figure 1(a–e) gives the Raman shifts calculated for cubic Si, CdS, InP, InAs and wurtzite CdSe nanocrystals along with the experimental data and the results from other models. The experimental results are in good agreement with our calculated values. From Fig. 1a, it can be seen that the phonon confinement model developed by Richter, Wang, and Ley (RWL)^4^ and the bond polarization (BP) model^6^ do not provide quantitative agreement between the Raman peak positions and the nanocrystal size, and they significantly underestimate the Raman shift of Si nanocrystals.

To further understand the role of surface effects, we may estimate the proportion of surface effect shift in Raman shift by: *η *= |*F*^surface^Δ*ω*(D)^surface^/Δ*ω*(*D*)|. The calculated *η* is about 10%, 20%, 30%, 30%, 40% and 80% for Si, CdSe, InP, InAs, diamond and CdS nanocrystals, respectively, as shown in Fig. 2. The *η* of Si nanocrystals are lowest. The *η* of diamond and CdS nanocrystals are higher. Thus, it can be concluded that the large shifts of first-order Raman spectra in Si nanocrystals mainly result from the quantum effect shifts. But the contribution of surface effect shift in nanodiamond is so great that cannot be ignored.

In order to measure changes in crystal size, RWL’s scaling relationship between the Raman peak position and nanocrystal is frequently used: *ω*(*D*) *− ω*(*∞*) = A(a_0_/*D*)^γ^, a_0_ is the lattice constant, A and γ are two adjustable parameters^4^,^6^. Raman spectroscopy is also recommended as a fast and powerful tool for determination of the size of nanocrystalline diamond (DND) produced by detonation synthesis^24^,^25^,^26^. The first-order Raman spectrum of a bulk diamond displays only one triply degenerated Raman line at *ω*(*∞*) = 1332 cm^−1^. The shift of the first order Raman peak of the small DND is lower by a few wavenumbers than the shift observed for the bulk diamond. The Raman peak of DND sample after oxidation in air at 430 °C for 6h is 1326 cm^−1^^25^. The calculated size of the DND using the RWL model is 6.4 nm, which is remarkable high value compared to XRD data, 5.2 nm^25^. Therefore, further theoretical work is needed to provide a quantitatively accurate model of the size dependence of the Raman spectra.

According to Eq. 8, the relationship between the number of carbon *N* and Raman frequency in DND can be written as


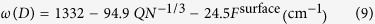


Because there is no adjustable parameter, Eq. 9 can be used to more accurate calculation for the size of DND. For example, when the measured *ω*(*D*) for the DND is 1323 cm^−1^ and 1326 cm^−1^ 24,26, the corresponding calculated size of DND using Eq. 9 is 3.2 nm and 5.0 nm, respectively. Results are in exceptional agreement with that measured by XRD, 3.2 nm and 5.2 nm.

In conclusion, a quantitatively accurate model of the size dependence of the Raman spectra of nanocrystals is developed. The theoretical curves of diamond, Si, CdS, CdSe, InP, InAs nanocrystals are computed without adjustable parameters. Results are in good agreement with the available experimental data. The empirical relationship between the Raman shift and size reported here for the diamond nanocrystals can provide a good formula for using Raman spectroscopy as a tool to measure size of nanocrystals. Due to the simplicity and applicability of the model, it may be extended to more other nanomaterials.

## Additional Information

**How to cite this article**: Gao, Y. *et al.* Size-Dependent Raman Shifts for nanocrystals. *Sci. Rep.* **6**, 20539; doi: 10.1038/srep20539 (2016).

## Figures and Tables

**Figure 1:**
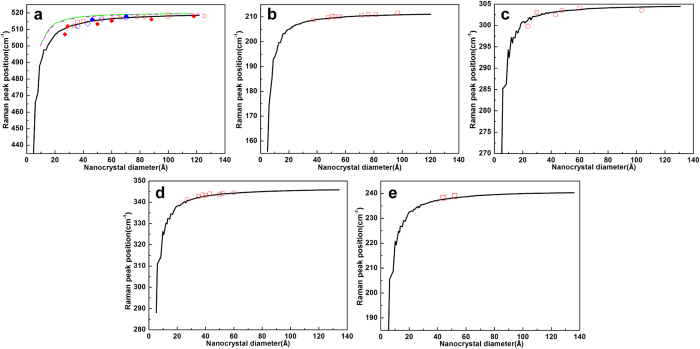
Raman peak position as a function of the nanocrystal diameter for zinc blend (**a**) Si, (**c**) CdS, (**d**) InP, (**e**) InAs, and (**b**) wurtzite CdSe nanocrystals. The blue solid lines are from our calculations of the spherical nanocrystals. The green thin line and magenta dot line are the predicted Raman peak positions versus size from the RWL and BP models, respectively. The open squares, circles and diamonds are the experimental data [a, refs 12,13,19, 20, 21 (open squares, open circles, open blue diamonds, solid blue diamonds, solid red diamonds); (**b**), ref. 22 (open squares); (**c**), refs 2,3 (open squares, circles); (**d**), refs 2,23 (open squares, circles); (**e**), ref. 2 (open squares)].

**Figure 2:**
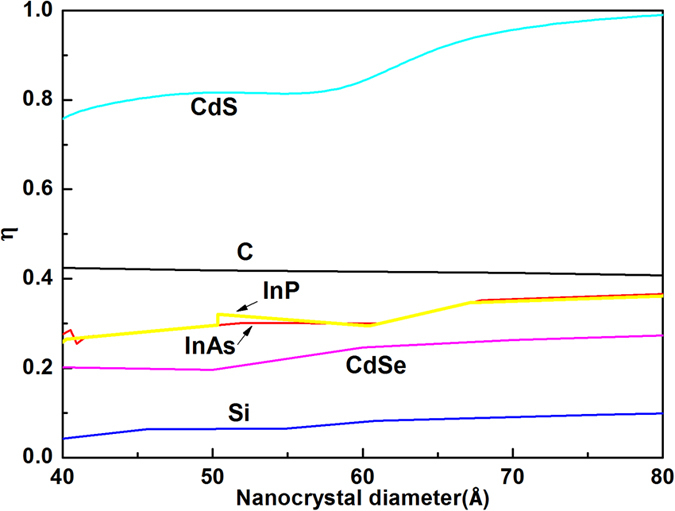
Ratio of surface bonding contribution in Raman red shifts.
